# MNISQ: A Large-Scale Quantum Circuit Dataset for Machine Learning in the NISQ Era

**DOI:** 10.1038/s41597-026-07493-9

**Published:** 2026-05-26

**Authors:** Leonardo Placidi, Ryuichiro Hataya, Toshio Mori, Koki Aoyama, Hayata Morisaki, Kosuke Mitarai, Keisuke Fujii

**Affiliations:** 1 Graduate School of Engineering Science, The University of Osaka, 1-3 Machikaneyama, Toyonaka, 560-0043 Osaka Japan; 2 Center for Quantum Information and Quantum Biology, The University of Osaka, 1-2 Machikaneyama, Toyonaka, 560-0043 Osaka Japan; 3 RIKEN Center for Advanced Intelligence Project (AIP), RIKEN, Nihonbashi 1-chome Mitsui Building, 15th floor, 1-4-1 Nihonbashi, Chuo-ku, 103-0027 Tokyo Japan; 4 RIKEN Center for Quantum Computing (RQC), RIKEN, Hirosawa 2-1, Wako, 351-0198 Saitama Japan

**Keywords:** Quantum simulation, Databases, Scientific data

## Abstract

We introduce MNISQ, the first large-scale dataset for both quantum and classical machine learning during the NISQ era, containing 4.95 million circuits of 10 qubits constructed with up to 100 two-qubit gates. MNISQ serves as a foundational resource for developing natural language processing (NLP) models for quantum computing and deep learning models. The dataset is derived from quantum-encoded classical data (e.g., MNIST) and is available in two formats: quantum circuits and classical descriptions (Quantum Assembly Language, QASM). We perform baseline experiments on circuit classification using both quantum and classical methods. Quantum Kernel methods achieve up to 97% accuracy in multiclass classification. We also explore the impact of noise in quantum machine learning, helping develop error-mitigation strategies for noisy hardware. In classical experiments, we use QASM files with NLP models: S4, Transformer, and LSTM. The S4 model reaches 77% accuracy (81% with data augmentation), demonstrating that modern machine learning models can effectively classify quantum circuits. The dataset is publicly available at 10.5281/zenodo.19656638  and related codes are available on GitHub.

## Background & Summary

### Background

Machine Learning (ML) is a discipline intricately shaped by the availability and quality of data^1^. Quantum computers, initially conceived for tackling complex physics problems due to their computational advantages^2^, have given rise to the development of “quantum” machine learning (QML) techniques^3,4^. However, the scarcity of quantum datasets remains a challenge, and many experiments within the community rely on custom data processing and evaluation methods rather than standardized benchmarks.

Contemporary quantum computers fall under the category of Noisy-Intermediate-Scale Quantum (NISQ) devices^5^. Ranging from a few qubits to a few hundred, these devices lack full error correction and operate within the domain of small-to-medium scale hardware prone to noise. Despite these present constraints, research avenues are diverse and oriented toward practical applications^3,6^. Quantum computers, characterized by their BQP complexity class^2^, hold the potential to excel beyond classical counterparts in various problem domains^7^. Consequently, they offer the promise of enhanced data processing capabilities and accelerated execution of machine learning tasks^4,8^. The ongoing research landscape has yielded noteworthy progress with quantum algorithms in supervised^4,9^, unsupervised^10,11^ and reinforcement learning^12–14^. However, the existing quantum devices mostly suffice for addressing small problems, preventing a direct comparison with cutting-edge classical machine learning techniques. Anticipated advancements in this decade project the ability of quantum devices to support considerably larger NISQ computations with thousands of qubits (e.g., the IBM Development Quantum Roadmap). Consequently, successful breakthroughs in quantum algorithms and machine learning methodologies could usher in transformative changes across artificial intelligence and all scientific domains. An important direction is the challenge to find scenarios where quantum methods present an advantage of the classical counterpart. Our work addresses the research of an advantageous framework with the task of quantum circuit classification.

The present work introduces MNISQ, a large scale quantum circuit dataset aimed at delivering a standard testing environment for the community. MNISQ can be easily employed for machine learning on quantum computers (e.g., quantum machine learning) and machine learning for quantum computers (as a support tool to perform some tasks without relying on quantum hardware). Along the introduction of the dataset, we also present quantum machine learning and classical machine learning baseline experiments to show the dataset learnability and compare the performance of the two approaches.

### Related Works

Here follow a series of existing studies concerning data associated with quantum systems, referred to as quantum datasets.

**NTangle**^15^: NTangle shares common ground with our research in the sense that it emphasizes the importance of focusing on quantum-related datasets to gain an advantage in QML. However, it defines the quantum state itself as the dataset, which, unlike our quantum circuit dataset, cannot be directly applied to existing classical machine learning.

**QDataSet**^16^: QDataSet is a dataset defined from 52 types of data concerning quantum operations for single and two-qubit systems, encompassing both noise-inclusive and noise-free scenarios. It is constructed to apply existing machine learning frameworks for characterizing quantum systems and improving experimental control setups including classical postprocesssing, and hence QDataSet constitutes a dataset pertaining to the physical dynamics of one- or two-qubit systems. Each dataset consists of 10, 000 data points.

**Quantum Federated Data**^17^: a quantum federated dataset which can be used for distributed learning in QC networks and can serve as a baseline for future quantum federated learning implementations with quantum sensors.

**Quantum datasets on PennyLane** PennyLane is an open-source software library that provides tools for quantum machine learning and quantum computing. Quantum Datasets in PennyLane, specifically for quantum chemistry and quantum spin systems provide detailed problem systems descriptions, parameterized models and their solution for these problems, aiding in the study of quantum algorithms related to these molecular and spin systems. While these datasets serve as a valuable baseline for transparent research, how to apply quantum and classical machine learning for these data remain challenges, the size of the dataset is also too small to be applied for the state-of-the-art machine learning techniques. Besides that, our dataset is also already partially available on PennyLane datasets page at https://pennylane.ai/datasets/mnisq.

**VQE-generated dataset**^18^: The VQE-generated dataset is the most akin to our approach. It is a quantum circuit dataset labeled by the computational task it performs. However, a significant issue arises due to the dataset’s size, as there are only 300 quantum circuits for each label, which is too small to apply state-of-the-art machine learning techniques.

MNISQ contains 4.95 million data points, with circuits of 10 qubits up to 100 two-qubit gates. This scale is several orders of magnitude larger than existing quantum circuit datasets for machine learning, which typically contain from a few hundred to at most a few thousand samples per task. For example, the VQE-generated dataset provides approximately 300 circuits per class, and QDataSet consists of multiple datasets of 10,000 samples each for small (1-2 qubit) systems.

#### Motivation and approach

The motivation behind this study is to present a framework of quantum circuit classification. In contrast to classical data classification, the availability of an encoded dataset allows comparing quantum and classical methods. While it has been theoretically shown that QML can be advantageous for certain tasks, there is a need to explore practical and experimentally friendly settings to showcase the potential of QML. One approach is to work with quantum data obtained from quantum systems directly, without converting them to classical formats, as it has been shown to be more efficient for certain learning tasks^7^. However, this approach presents challenges in transferring quantum states obtained from physical experiments to quantum computers, making it experimentally challenging. Another approach is to define artificial machine learning tasks that are solvable only by quantum computers, such as the discrete logarithm problem^19^. While this demonstrates a rigorous advantage of QML, it is in a highly artificial setting and may not directly translate to advantages in practical problems. Recent work has also suggested that practical quantum advantage may emerge in physics-related machine learning tasks, for example in ghost imaging, where hybrid quantum-classical models achieved improved performance over comparable classical methods in identification and imaging tasks^20^. To address these limitations and establish a more practical and experimentally friendly setting, it is crucial to create datasets where the advantage of QML can be expected and compare its performance against classical approaches. Our motivation is to propose a new machine learning framework that analyzes and learns from “quantum circuit data” given as input. Quantum computing is still in its infancy, but as quantum computing becomes more widely used, more quantum circuit data will be accumulated in the world. Just as ChatGPT^21^ and other software can already handle programming languages and generate optimized code, learning quantum circuit data, analyzing it, and generating optimized quantum circuits will be a very important field where we can apply machine learning. Many quantum circuits have already been run using AWS and IBM cloud systems, and quantum circuit data is stored on the servers, but unfortunately we do not have access to that data. What is needed to accelerate the framework of learning from such quantum circuit data, is to define a large amount of quantum circuit data sets and start machine learning research using them. We have not developed a method for MNIST classification, but rather we have generated quantum circuit datasets with AQCE^22^ using MNIST data as a seed and have launched a completely new framework for machine learning on quantum circuit datasets.

This dataset is provided in a dual form that can be utilized for both quantum and classical machine learning. The scale and structure of MNISQ enable several concrete research directions beyond existing quantum datasets. In particular, MNISQ supports the systematic investigation of the scalability of QML models with respect to data size and circuit complexity, as well as the study of sample complexity in hybrid quantum-classical learning settings. Furthermore, the availability of both quantum circuit representations and classical descriptions (QASM) provides a controlled testbed for developing and benchmarking “quantum-aware” classical models that learn from structured quantum data. These research directions are difficult to explore with existing small-scale datasets and position MNISQ as a benchmark for large-scale studies in quantum machine learning.

**Fig. 1:**
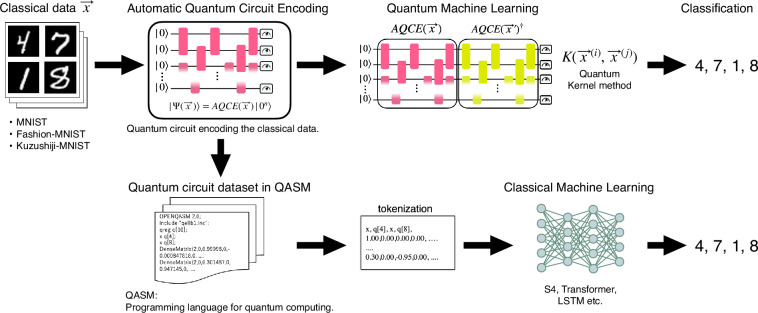
Summary of the workflow for this study. The AQCE algorithm generates a quantum circuit that embeds classical data $\vec{x}$ into a quantum state $\left|\Psi \right\rangle =AQCE(\vec{x})\left|{0}^{n}\right\rangle$ and delivers a dataset in dual form. Quantum machine learning (quantum kernel method) and classical machine learning (LSTM, S4, Transformer) are applied to classify the data the quantum circuits or qasm files originated by the encoding procedure.

The workflow of this study is summarized in Fig. 1.

### Contributions

- We introduce a large-scale quantum dataset in the form of quantum circuits and QASM, facilitating research in quantum machine learning and classical machine learning. The dataset is easily accessible on Zenodo at 10.5281/zenodo.19656638^23^, and, given its QASM formalism, easily used in Qulacs, Qiskit, PennyLane.
- We introduced a new framework where quantum and classical machine learning learn (and compete) on quantum circuit data.
- We present a quantum machine learning baseline, also investigating the effect and mitigation of noise.
- We explore the classification of circuits using classical models (S4, Transformers, LSTM), revealing the solvability of the problem by classical machine learning. We highlight the remarkable capability (and the implications) of classical machine learning in comprehending quantum circuit computations without prior knowledge of quantum mechanics.
- We introduced a task of quantum circuit classification where quantum machine learning methods are advantageous compared to classical methods.

## Methods

### MNISQ Dataset: Construction and Characteristics

**Fig. 2:**
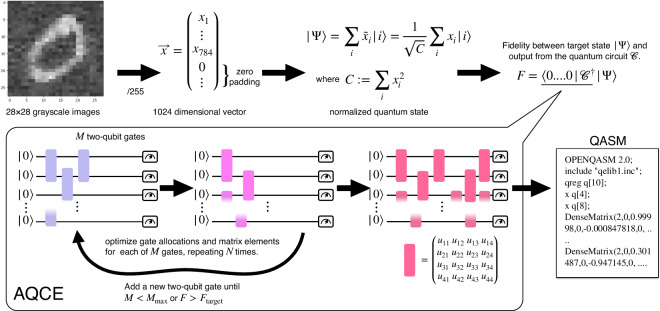
Schematic diagram of how to embed classical data into the probability amplitude of a quantum state using the Automatic Quantum Circuit Encoding (AQCE)^22^ algorithm.

To create a quantum circuit dataset, we start from classical data like MNIST. The transformation of the classical data into quantum states is a pivotal task and we utilize the Automatic Quantum Circuit Encoding (AQCE) approach^22^. Figure 2 illustrates the procedure.

The literature^4,9,24^ explored many kinds of encodings such as Basis Encoding, Amplitude Encoding, Repeated Amplitude Encoding, Rotation Encoding, Coherent State Encoding. Amplitude Encoding. Encoding information is a crucial task and can highly affect trainability (such as barren plateau^24^) for quantum machine learning models. AQCE is an algorithm to automatically generate an optimal circuit for the quantum encoding of information. It is a NISQ method, and it does not rely on parametric circuit optimization. AQCE is a NISQ algorithm and difficult to scale with the number of qubits, but our work resides in the NISQ dataset generation, so AQCE is a good choice for an encoding strategy.

### Quantum Encoding of Classical Information (AQCE method)

Given classical data $\vec{x}$ as *d*-dimensional vector, AQCE generates a quantum circuit ${\mathcal{C}}$ that encodes $\vec{x}$ into the complex amplitudes of the output quantum state:

1 $$\begin{array}{r}\left|\Psi \,\right\rangle :=\sum _{i\in {\{0,1\}}^{n}}{\widetilde{x}}_{i}\left|i\right\rangle ,\end{array}$$

where the number of qubits *n* is chosen to be $n:=\lceil {\log }_{2}(d)\rceil$ and the complex amplitudes ${\widetilde{x}}_{i}$ are normalized appropriately so that ${\sum }_{i}| {\widetilde{x}}_{i}{| }^{2}=1$. If 2^*n*^ > *d*, for those indices that do not have corresponding elements in $\vec{x}$, ${\widetilde{x}}_{i}$ is set to be zero. AQCE constructs such a quantum circuit ${\mathcal{C}}$ generating any given quantum state $\left|\Psi \right\rangle$ by optimizing the configuration and parameters of the quantum gates in ${\mathcal{C}}$ by maximizing the absolute value of the following overlap, which we call fidelity here:

2 $$F=| \left\langle 0\right|{{\mathcal{C}}}^{\dagger }\left|\Psi \right\rangle | .$$

The AQCE iteration ends when the fidelity exceeds the target fidelity *F*_target_ or the maximum number of gates ${M}_{\max }$ initially set for ${\mathcal{C}}$. The resulting quantum circuit generated by the AQCE procedure from classical data $\vec{x}$ will be referred to as the $AQCE(\vec{x})$ circuit.

AQCE is intended to be performed by simulating a quantum computation on a classical computer. Although this can only be done on the order of tens of qubits, we believe that the dimension of the classical data that can be amplitude embedded is sufficient, for example, 10^9^ for a classical simulation of 30 qubits. Also, the embedding operation with AQCE may cause a computational bottleneck, but once the quantum circuit data is generated in this format (like JPG format for images), it can be used efficiently thereafter. For a more detailed description of AQCE, please refer to the supplementary material paragraph on the AQCE method.

### Dataset Construction

Using a cluster machine, we employed the AQCE method to encode the standard machine learning datasets MNIST^25^, Fashion-MNIST^26^, and Kuzushiji-MNIST^27^. These datasets were obtained from OpenML^28^. These datasets are publicly available and distributed under terms that allow reuse for research purposes.

The datasets are accessible via OpenML: MNIST, Fashion-MNIST, Kuzushiji-MNIST, and OpenML. Original dataset sources are also available at: MNIST, Fashion-MNIST, and Kuzushiji-MNIST. All source datasets used in this work are publicly available and distributed under permissive licenses that allow their use and redistribution for research purposes. The datasets were accessed via OpenML, which provides guidance on dataset usage and licensing.

For each dataset, we generated 60,000 training samples and 10,000 test samples. Additionally, to better train classical machine learning models, we augmented^29,30^ our quantum dataset to include 480,000 samples. The dataset strictly follows the original train/test splits of the source datasets. Data augmentation is applied exclusively to the training set, and no augmented samples derived from training images are included in the test set. Each fidelity variant (e.g., 80%, 90%, 95%) is generated independently from the same underlying train/test partition. While these variants correspond to different quantum circuit realizations of the same classical inputs, the separation between training and test data is preserved within each variant, and no sample or its derivatives appear across partitions. Classical training data were augmented offline from the original 60,000 training images using a three-stage sequential pipeline. First, we added one rotated variant per image using a fixed rotation angle of +50 degrees. Second, we added one crop variant per image using RandomCrop(size=(28,28)). Third, we added one shifted/affine variant per image using RandomAffine with degrees=(0,30), translate=(0.1,0.3), and scale=(0.5,0.75). Because each stage appends one additional transformed sample per current sample, dataset size doubles at each stage: 60,000  → 120,000  → 240,000  → 480,000, yielding 480,000 training samples for each dataset.

We created three variations for each dataset. Variations correspond to different generation procedures with a minimum fidelity of 80%, 90%, and 95% for the generated quantum circuit dataset. Higher fidelity values require a larger number of quantum gates in the circuit.

## Data Records

The MNISQ dataset is publicly available on Zenodo at 10.5281/zenodo.19656638^23^.

**Fig. 3:**
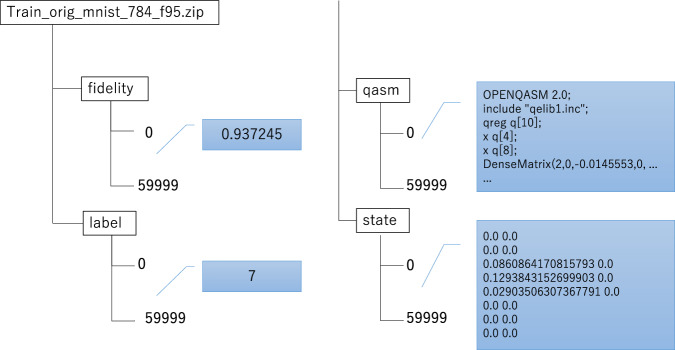
Example of the directory structure of one MNISQ archive, showing the stored fidelity values, class labels, QASM files, and target state vectors for each encoded sample.

As illustrated in Fig. 3, each archive stores one subdataset together with the information required to reconstruct and reuse the encoded samples. MNISQ is organized into subdatasets defined by three factors: source dataset, fidelity target, and representation format. The source datasets are MNIST, Fashion-MNIST, and Kuzushiji-MNIST. For each of them, three fidelity levels are provided (80%, 90%, and 95%). In addition, both original and augmented training sets are available, together with test sets and two QASM representations.

Overall, MNISQ contains 9 original subdatasets (three source datasets × three fidelity levels), each with 60,000 training samples and 10,000 test samples, together with 9 augmented training subdatasets containing 480,000 training samples each. The total size is therefore 4,950,000 records. QASM files are distributed in two formats: *QASM with DenseMatrix formalism*, intended for Qulacs, and *base QASM formalism*, where the proprietary DenseMatrix operator is decomposed into standard gates for compatibility with platforms such as Qiskit and PennyLane.

Each encoded sample is associated with the following stored records:

- **fidelity** the fidelity between the AQCE-generated circuit output state and the target amplitude-encoded state;
- **label** the class label inherited from the original image dataset;
- **qasm** the quantum circuit description in QASM format;
- **state** the target state vector corresponding to the encoded classical input.

File and archive names follow a structured convention of the form:


[data]_[type]_[fidelity].zip


where data identifies the split and representation, type identifies the source dataset, and fidelity identifies the AQCE fidelity target.

### Details on dataset generation

This section summarizes the parameters and hardware used for generation of the MNISQ records.

Table 1 reports the AQCE generation parameters for each target fidelity. Here, *M* denotes the maximum number of two-qubit gates allowed in the generated circuit, while *δ* determines how many gates are added at each AQCE iteration.

**Table 1:** Parameters used to achieve the target fidelity in AQCE generation.

Fidelity (%)	Max. 2-qubit gates *M*	Increment *δ*
80	25	3
90	50	6
95	100	12

The dataset was generated on a cluster machine with the following specifications:

- CPU: Dual Intel Xeon Platinum 9242 (2.3 GHz, 48-core)
- Memory: 384 GB (24  × 16 GB DDR4-2933 Reg. ECC)

Generating one MNIST sample at 90% fidelity required approximately 8 seconds per process. Although AQCE can be executed with multithreading, we adopted single-threaded execution because it was more efficient for large-scale parallel generation.

For augmented datasets, 480,000 samples were generated by distributing the work across 1000 independent processes, each producing 480 samples. Each process was submitted as a single job to the cluster. To reduce disk I/O contention, each process accumulated its 480 generated circuits in memory and wrote them to disk only after completion. Using a 40-node cluster (3840 cores), the full 4.95 million-record dataset was generated in approximately 8.5 hours.

Table 2 summarizes the source datasets included in MNISQ. For each source dataset, original train/test splits are provided together with augmented training data and all three fidelity targets.

**Table 2:** Breakdown of the MNISQ records by source dataset.

Dataset	Original train/test	Augmented train	Fidelity levels
MNIST	60,000 / 10,000	480,000	80, 90, 95
Fashion-MNIST	60,000 / 10,000	480,000	80, 90, 95
Kuzushiji-MNIST	60,000 / 10,000	480,000	80, 90, 95

### Dual-form representation: quantum circuits and QASM descriptions

Quantum circuits for Qulacs can be loaded using methods of the form:


Qulacs_dataset.[type].loader.load_[type]_[data_type]_[fidelity]


In this notation:

- **type** specifies the source dataset: mnist, fashion_mnist, or kuzushiji_mnist;
- **data_type** specifies the split: original training, augmented training, or test;
- **fidelity** specifies the AQCE target fidelity: f80, f90, or f95.

For example, the MNIST training dataset with target fidelity 95% can be loaded as:


Qulacs_dataset.mnist.loader.load_mnist_train_f95()


Executing one stored circuit embeds the corresponding classical input into a quantum state. This state can then be used directly as input to downstream quantum machine learning pipelines.

The Qulacs-oriented QASM representation uses the proprietary DenseMatrix instruction to encode gate blocks compactly. Since DenseMatrix is not part of standard OpenQASM, a second representation is also provided in which these operations are decomposed into standard gates for compatibility with Qiskit and PennyLane.

The DenseMatrix instruction has the form:

DenseMatrix(number of target qubits, number of control qubits, matrix elements a + bi ordered as “a, b”), target qubit column, control qubit column;

An example is:

DenseMatrix(2,0,0.900726,0,-0.267421,0,...,0.752815,0) q[2],q[6];

Within each archive, the directory structure is organized by record type:

- **fidelity** fidelity values for all encoded samples;
- **label** labels associated with each encoded sample;
- **qasm** QASM circuit files;
- **state** target state vectors corresponding to the encoded samples.

The main archive prefixes are:

- train_orig 60,000 original encoded training samples in QASM with DenseMatrix;
- base_train_orig the same original training samples in base QASM without DenseMatrix;
- train 480,000 augmented training samples in QASM with DenseMatrix;
- test 10,000 encoded test samples in QASM with DenseMatrix;
- base_test the same test samples in base QASM without DenseMatrix.

The dataset identifiers are:

- mnist_784 MNIST;
- Fashion-MNIST Fashion-MNIST;
- Kuzushiji-MNIST Kuzushiji-MNIST.

The fidelity suffixes are:

- f80 fidelity ≥80%;
- f90 fidelity ≥90%;
- f95 fidelity ≥95%.

In addition to enforcing fidelity thresholds during AQCE generation, we carried out practical validation of the generated records by checking that the stored circuit representations reproduce the intended encoded states and associated source images upon execution. To support portability, the dataset includes both a Qulacs-oriented QASM representation using DenseMatrix and a base QASM representation intended for compatibility with other frameworks such as Qiskit and PennyLane. The integrity and usability of these files are further supported by the public tutorials and examples distributed with the dataset repository, as well as by subsequent reuse of MNISQ in the community.

Accordingly, archives such as test_mnist_784_f90.zip or base_train_orig_Fashion-MNIST_f95.zip can be interpreted directly from their filenames. All such archives are available through the Zenodo repository.

## Technical Validation

### Baseline for MNISQ

In this section, we present a baseline with quantum models and classical models. Our experiments serve to show the challenges depending on the data representations, indicating a relative advantage for quantum models in this scenario of circuit classification. In particular, the quantum representation directly encodes amplitudes of the target quantum state, while the classical representation corresponds to a syntactic description of the circuit. As a result, the two settings reflect different learning problems.

### Quantum Machine Learning experiments

In the natural task of multiclass classification, MNISQ can be investigated by explicit (such as variational algorithms^3,31^) and implicit quantum models (such as quantum kernel^32^). In these first experiments, we chose to present results based on the Quantum Kernel approach. The use of a quantum kernel is motivated by its ability to compute classically hard or intractable kernels^9,32,33^, the existing literature for classification tasks^34,35^, along with their relative efficiency with parallel computation on simulators. In addition, recent studies have shown that quantum kernels can offer a quantum advantage over classical methods on carefully engineered datasets^19^ representing a very promising direction for the quantum community^8^.

In the literature, the classification of MNIST and image datasets has been explored in the variational^36–38^, kernel^35^ and tensor networks^39^ literature in different ways. Among the frameworks, the most promising is the quantum convolutional network^40^ with already significant works in the quantum classification field^38,41^. Existing methods are generally based on one or multiple encoding of the data (often rescaled to fit less qubits) and, in limited cases, in the adoption of classical models in part of the pipeline^42–44^. To the best of our knowledge, the existing classification of encoded data is mostly based on binary classification or 4-class classification, so we believe that the 10 classes of MNISQ may also present an interesting direction for future works on variational algorithms.

#### Quantum Support Vector Machine (QSVM)

The quantum experiments are based on QSVM, so we shortly introduce the main idea and leave in the supplementary material an introduction to the classical Support Vector Machine (SVMs), which differ from QSVM only in the computation of the kernel while leaving the same optimization procedure.

In fact, QSVMs differ from SVMs only because in the formulation of the decision function:

3 $$f(\vec{x})={\rm{sgn}}\left(\mathop{\sum }\limits_{n=1}^{N}{t}_{n}{\alpha }_{n}K({\vec{x}}^{(i)},\vec{x})\right),\vec{x}\in {\rm{TestSet}},$$

**Fig. 4:**
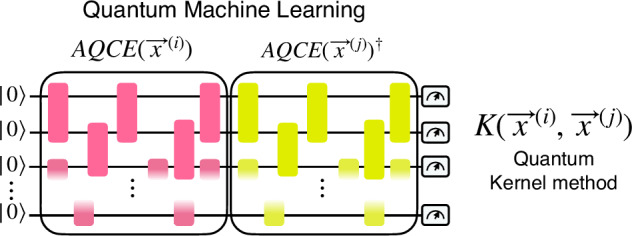
Example of the standard computation of the quantum kernel elements by generating the circuits $AQCE({\vec{x}}^{(1)})AQCE{({\vec{x}}^{(2)})}^{\dagger }$ for every pair of elements and computing the probability of the zero state after measurement in the computational basis.

The Kernel function $K\left({\vec{x}}^{(i)},{\vec{x}}^{(j)}\right)$ is obtained by taking the inner product between quantum states, as detailed in Fig. 4. In our examples, the quantum states come from the encoding of our information (AQCE) available in the dataset, thus we can also write our quantum kernel as:

4 $$K\left({\vec{x}}^{(i)},{\vec{x}}^{(j)}\right)=| \left\langle 00...0\right|AQCE({\vec{x}}^{(i)})AQCE{({\vec{x}}^{(j)})}^{\dagger }\left|00...0\right\rangle {| }^{2}$$

#### QSVM implementation details

We use a support vector classifier with precomputed kernel (scikit-learn) with regularisation parameter *C* = 1.0 and no additional hyperparameter tuning.

In the ideal (noise-free) setting, kernel elements are computed via statevector simulation, allowing construction of the full kernel matrices on the dataset splits used in the experiments.

**Fig. 5:**
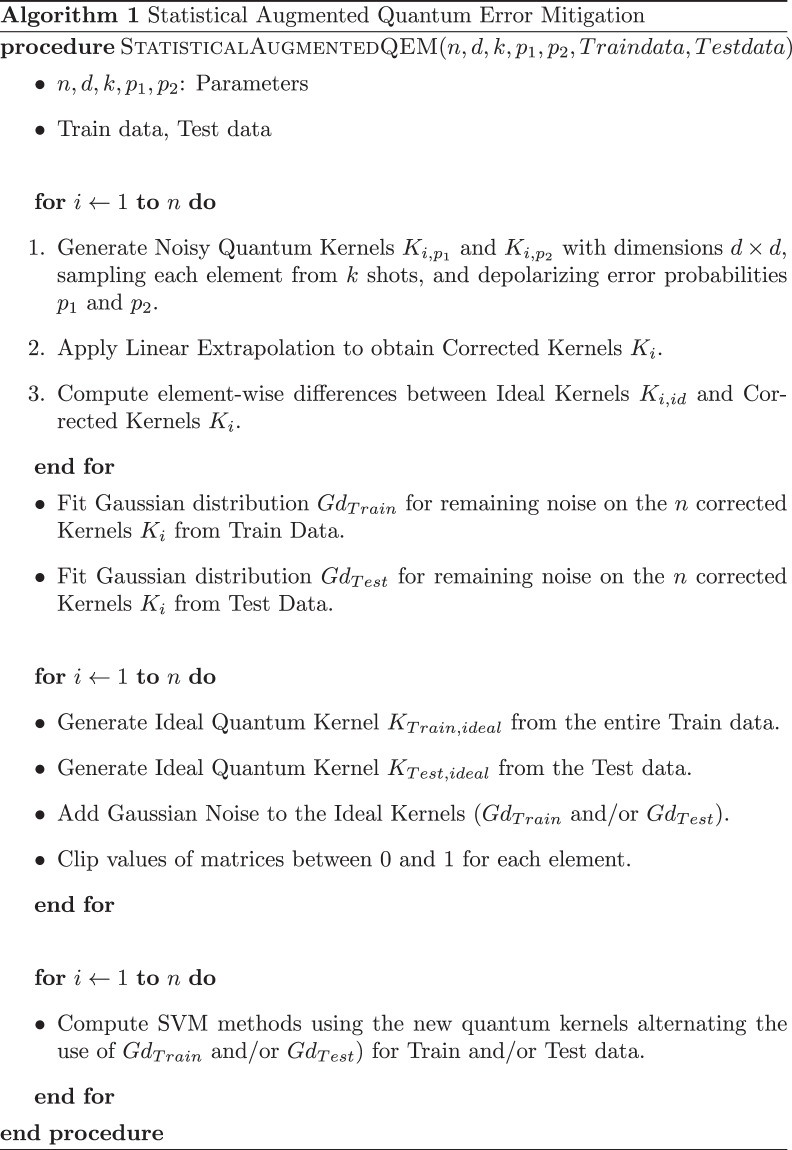
Algorithm to obtain an approximate residual noise after mitigation of a noisy run over MNIST_784 dataset.

In contrast, in the noisy setting, each kernel element must be estimated via repeated circuit executions (shots) and error mitigation. Due to the resulting computational cost and the *O*(*N*^2^) scaling of kernel methods, full kernel evaluation was infeasible on our devices. Therefore, all noisy experiments are conducted on reduced subsets of size *d* = 200, using *k* = 10, 000 shots per circuit and *n* = 10 repeated simulations, as detailed in Fig. 5. In the ideal (statevector) setting, the QSVM evaluation is deterministic and does not exhibit variability across runs. In contrast, in the noisy setting, results are stochastic due to sampling, and variability is reported via standard deviations.

We compare our prediction results based on the accuracy of the decision function and the results are given in multiclass classification accuracy.

#### Quantum Baseline

Table 3 shows our results on the three subdatasets of MNISQ (mnist_784, Fashion-MNIST, Kuzushiji-MNIST). The two methods employed are a variation of the quantum support vector machines (QSVMs) where, instead of performing a binary classification task, we perform a multiclass classification.

**Table 3:** Experiments with the quantum kernel approach in Qulacs under ideal statevector simulation.

Dataset	Fidelity	1-versus-1 QSVM	1-versus-the-rest QSVM
MNIST-784	80	0.9486	0.9494
	90	0.9733	0.9713
	95	**0.9791**	0.9777
Fashion-MNIST	80	0.8204	0.8151
	90	0.8541	0.8463
	95	**0.8678**	0.8656
Kuzushiji-MNIST	80	0.8417	0.8360
	90	0.8909	0.8847
	95	**0.9066**	0.9004

Looking at Table 3, the *one-versus-one* approach fits $\frac{{n}_{{\rm{classes}}}({n}_{{\rm{classes}}}-1)}{2}$ QSVMs on all the possible couples of classes and finally classifies based on a voting scheme called *one-versus-one*. On the other side, *one-versus-the-rest* approach consists in training *n*_*classes*_ QSVMs (10 for MNISQ subdatasets), each one able to classify one class versus any other. We performed our experiments using Qulacs^45^ quantum computer simulator for the quantum kernels and sklearn implementations for the classifiers^46^. The *one-versus-the-rest* training is significantly faster because of its limited number of classifiers^47^, but since every classifier is trained on a small subset of the dataset, the *one-versus-one* is likely more robust and, in fact, presents a slightly better performance. We observe that our best prediction is reached when the fidelity is at least 95% for the data. Thus, a higher fidelity circuit leads to a better approximation of the target state and a more effective feature map for the quantum kernel.

#### Performance of QML under noisy operations

The MNISQ dataset is a 10-qubit quantum circuit with a total number of quantum gates of about 100, which should be sufficient to run on a state-of-the-art quantum computer achieving quantum volume 2^19^. To demonstrate this, we introduce 1- and 2-qubit depolarizing noise after each gate with error probability *p* = 0.001 or *p* = 0.002, and estimate the kernel elements by sampling with error mitigation via zero-noise extrapolation (ZNE) using linear extrapolation^48,49^. Depolarizing noise is a standard and widely used effective noise model in NISQ studies, as it provides a simple way to capture generic gate imperfections and loss of coherence without assuming device-specific calibration details.

Given that the datasets have 60, 000 training samples and 10, 000 test samples each, it is not feasible to compute the full noisy kernel matrix. The main bottleneck is that each circuit must be executed with a large number of shots (thousands to millions), resulting in runtimes of approximately one minute per circuit on our machines. Therefore, in the noisy setting we construct kernel matrices on subsets of size *d* = 200, using *k* = 10, 000 shots per circuit, *n* = 10 repeated simulations, and analyze the average residual noise after mitigation. We perform this study on the three dataset versions and report standard deviations due to the stochastic nature of the experiments.

In our results, the Train/Train, Test/Test, and Train/Test configurations correspond to different combinations of noise distributions applied to the kernel matrices. In the Train/Train and Test/Test settings, the same noise distribution is used for both training and testing, while Train/Test introduces a mismatch between the two.

We described the detailed algorithm in Fig. 5.

In Table 4 we show the distributions of the residual noise after mitigation for the MNISQ_784 dataset with different fidelities. Due to the limited number of shots, the residual noise can be well approximated by Gaussian distributions. As shown, the noise fitted on the training data exhibits a lower standard deviation ( ~ 0.008) compared to the test data (~0.150). This effect could be mitigated by increasing the number of simulations, assuming consistent noise settings across runs.

**Table 4:** Summary of train and test statistics.

Statistic	MNIST_784_f80	MNIST_784_f90	MNIST_784_f95
Train Mean	0.0004554	0.0016718	0.0054822
Standard Deviation (Train)	0.0079759	0.0081285	0.0085336
Test Mean	0.0074991	0.0090838	0.0141085
Standard Deviation (Test)	0.1508941	0.1553731	0.1587207

The reported standard deviations quantify the variability introduced by sampling, and this variability propagates to the observed differences in classification accuracy.

Finally, the accuracy scores for the QSVM obtained with the above statistical mitigation procedure are reported in Table 5. In the Train/Train configuration, the noise distribution fitted on the training data is used to perturb both training and test kernels. In Test/Test, the distribution fitted on the test data is used for both. In Train/Test, the training kernels are perturbed using the training noise distribution, while the test kernels use the test noise distribution, introducing a distribution shift.

**Table 5:** Accuracy results applying Algorithm 1.

Dataset	Fidelity	Noise Statistic	1-vs-1 QSVM Accuracy (Train/Test splits)	1-vs-rest QSVM Accuracy(Train/Test splits)
MNIST-784	80	Train/Test	0.9583 / 0.4039	0.93505 / 0.2486
	90	Train/Test	0.9798 / 0.4672	0.96505 / 0.2801
	95	Train/Test	0.9821 / 0.4778	0.9689 / 0.2981
MNIST-784	80	Train/Train	0.9575 / 0.9435	0.93505 / 0.9349
	90	Train/Train	0.9796 / 0.9697	0.96505 / 0.9646
	95	Train/Train	0.9820 / 0.9755	0.9689 / 0.9713
MNIST-784	80	Test/Test	0.7290 / 0.8466	0.6824 / 0.8139
	90	Test/Test	0.7958 / 0.8955	0.7561 / 0.8745
	95	Test/Test	0.8100 / 0.8991	0.7727 / 0.8917

Here, *n* = 10 denotes the number of simulations, *d* = 200 the number of items per simulation, *k* = 10.000 the number of shots per circuit, and *p*_1_ = 0.001, *p*_2_ = 0.002 the depolarizing noise parameters. Experiments were performed in Qulacs.

This mismatch explains the drop in accuracy observed in the Train/Test setting. When the noise distribution is consistent and concentrated (Train/Train), performance remains close to the ideal case, while larger variance in the noise (Test/Test) leads to a moderate degradation. These results indicate that the mitigated kernel remains competitive with the ideal kernel when the residual noise variance is sufficiently small.

### Classical Machine Learning baseline

We evaluate the performance of classical machine learning methods in recognizing quantum circuits using classical deep neural networks by treating the problem as sequence classification. Specifically, we used Transformer^50^, LSTM^51^, and Structured State Space (S4)^52^ models as sequence classifiers, which are trained to classify QASMs (Quantum Assembly Language) in the MNISQ datasets. While the literature presents examples of hybrid quantum-classical models^42,53^, our approach is a new direction where data and models are purely classical for the classification of quantum circuits. Detailed experimental settings are described in the supplementary material.

**Fig. 6:**
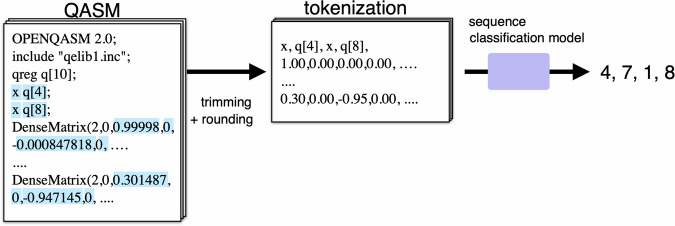
A schematic view of data processing in classical machine learning models.

The input QASMs were processed by removing unnecessary information, such as headers, and rounding dense matrix elements, e.g., from 0.12345 to 0.12, as shown in Fig. 6. We specify that we performed the experiments using the version of the dataset with QASM files with the proprietary extension *Dense()*.

Table 6 presents test accuracy of the classical machine learning models on the MNISQ datasets. S4 achieved the highest accuracy, with a test accuracy of 77.78% on mnist_784 with a fidelity of 95. This performance surpassed that of the other models by a significant margin. Interestingly, as the fidelity decreased, both the accuracy of the quantum methods and the accuracy of the S4 and LSTM also dropped. On the other hand, the accuracy of the Transformer showed the opposite trend, where datasets with lower fidelity resulted in better performance. Because higher-fidelity datasets have more information and consist of longer QASM sequences, these results suggest that S4 and LSTM can effectively capture long-range dependencies while Transformer cannot. In particular, the S4 model was designed to capture long-range dependencies^52^, and thus, achieved the highest performance.

**Table 6:** Test multiclass accuracy of classical machine learning models.

Dataset	Fidelity	S4	Transformer	LSTM
MNIST-784	80	66.61	43.42	59.06
	90	72.41	41.85	60.88
	95	**77.78**	40.40	66.93
Fashion-MNIST	80	64.93	46.22	59.30
	90	67.91	43.71	63.19
	95	**71.42**	43.60	64.64
Kuzushiji-MNIST	80	42.27	27.66	32.31
	90	46.64	26.87	37.79
	95	**51.83**	26.63	40.07

Table 7 shows test accuracy of the S4 model trained with and without augmented datasets. We can observe clear performance improvement on each dataset, indicating the effectiveness of data augmentation in MNISQ as in other machine learning problems^30^. We may also expect that dataset size matters to machine learning methods for quantum-circuit recognition tasks from these results.

**Table 7:** Test multiclass accuracy of the S4 model trained with and without augmented data at fidelity 95.

Setting	M-784	F-MNIST	K-MNIST
w/ aug.	**81.36**	**75.49**	**57.67**
w/o aug.	77.78	71.42	51.83

It is important to note that these results were achieved without incorporating any prior knowledge of quantum computing into the classical machine learning methods. Incorporating such knowledge could further improve the performance of these classical models in classifying quantum circuits.

In conclusion, MNISQ enables systematic studies with variational and gradient-based quantum models, hybrid quantum-classical approaches, and classical sequence models. It provides a controlled benchmark for analyzing robustness to noise and circuit complexity, evaluating error mitigation techniques, and developing data-driven methods to model noise effects in quantum circuits. While not designed for direct implementation of quantum error correction codes, the dataset can support benchmarking of noise-aware methods and preprocessing strategies relevant to error correction and fault-tolerant workflows. Given its scale and dual representation, the dataset also supports tasks such as circuit classification, generation, and quantum architecture search.

## Supplementary information

Supplementary Information

## Data Availability

The MNISQ dataset is publicly available via a persistent Zenodo repository at 10.5281/zenodo.19656638^23^. The dataset is also accessible via https://aqora.io/datasets/leopla/mnisq and https://pennylane.ai/datasets/mnisq.

## Code availability

Code, documentation and usage are accessible on GitHub at https://github.com/FujiiLabCollaboration/MNISQ-quantum-circuit-dataset, where README file, tutorials, and installation with PyPI are openly available. Version information for the baseline software environment is recorded in the repository dependency files (pyproject.toml and poetry.lock), including Python, NumPy, Scikit-learn, and Qulacs versions used for the reported experiments. The dataset is also hosted on Aqora (https://aqora.io/datasets/leopla/mnisq) and partially on PennyLane (https://pennylane.ai/datasets/mnisq).
