# Supplementary material for: MNISQ: A Large-Scale Quantum Circuit Dataset for Machine Learning in the NISQ Era
Source: Sci Data. 2026 May 26;13:810. doi: 10.1038/s41597-026-07493-9 (PMC13226669; doi:10.1038/s41597-026-07493-9)
Supplement: Supplementary file 1 — Supplementary Information [file 41597_2026_7493_MOESM1_ESM.pdf]

Supplementary Material for: MNISQ: A Large-Scale Quantum Circuit Dataset for Machine Learning in the NISQ Era

Leonardo Placidi, Ryuichiro Hataya, Toshio Mori, Koki Aoyama, Hayata Morisaki, Kosuke Mitarai, Keisuke Fujii

1 Quantum Computing Concepts

1.1 Basics of quantum computing

In this section, we elucidate the fundamentals of quantum computation and quantum gates, introducing the notation employed in quantum information. Contrary to classical information, which is represented by binary bits $\{0, 1\}$, quantum information defines the quantum bit, or qubit, as the smallest unit of information. This is represented as a linear combination of two orthogonal vectors, thus forming a superposition state:

$$|\psi\rangle = \alpha|0\rangle + \beta|1\rangle. \tag{1}$$

We utilize Dirac's bra-ket notation to describe column vectors using $|\cdot\rangle$ symbol:

$$|0\rangle = \begin{pmatrix} 1 \\ 0 \end{pmatrix}, \quad |1\rangle = \begin{pmatrix} 0 \\ 1 \end{pmatrix}. \tag{2}$$

Furthermore, the coefficients of the linear combination, referred to as complex probability amplitudes, are normalized to satisfy $|\alpha|^2 + |\beta|^2 = 1$. In addition to the column vector $|\psi\rangle$, we define the row vector obtained by taking the complex conjugate and transpose, or adjoint, of this:

$$\langle\psi| = \alpha^*\langle 0| + \beta^*\langle 1| = (|\psi\rangle)^\dagger. \tag{3}$$

where

$$\langle 0| = (|0\rangle)^\dagger = (1, 0), \quad \langle 1| = (|1\rangle)^\dagger = (0, 1). \tag{4}$$

Then, inner product of two vectors $|\psi\rangle, |\phi\rangle$ can be expressed as $\langle\phi|\psi\rangle$. Also, a linear operator can be represented by $|\phi\rangle\langle\psi|$.

For such a qubit, the operation executable on a quantum computer, the quantum gate, is specified by a 2×2 unitary matrix:

$$\mathbf{U} = \begin{pmatrix} u_{0|0} & u_{0|1} \\ u_{1|0} & u_{1|1} \end{pmatrix}, \tag{5}$$

The reason for being limited to unitary matrices is to preserve the normalization condition. The symbol $|$ in-between the index is to explicitly show which left and right indices correspond to output and input, respectively.

When describing multiple qubits, we consider the multiple tensor product space of a qubit space as a unit. As a basis for the tensor product space by n qubits, the direct product state of $\{|0\rangle, |1\rangle\}$ specified by the n-bit binary bit string $i_1 ... i_n$,

$$|i_1 .... i_n\rangle = |i_1\rangle \otimes \cdots \otimes |i_n\rangle \tag{6}$$

can be used. These 2^n orthonormal basis states are linearly combined to describe the quantum state of n qubits using 2^n complex numbers:

$$|\psi\rangle = \sum_{i_1 \dots i_n} c_{i_1 \dots i_n} |i_n\rangle \tag{7}$$

Hence, the quantum state of n qubits is represented as a 2^n-dimensional complex vector. Similar to the case of a single qubit, the complex vector satisfies the normalization condition:

$$\sum_{i_1 \dots i_n} |c_{i_1 \dots i_n}|^2 = 1. \tag{8}$$

When a quantum gate acts on the k-th qubit among n qubits, its action is:

$$U_k = I^{\otimes k-1} \otimes \mathbf{U} \otimes I^{\otimes (n-k)} = \sum_{i_1 \dots i_n} \sum_{j_k} u_{i_k|j_k} |i_1 ... i_k ... i_n\rangle\langle i_1 ... j_k i_{k+1} ... i_n|. \tag{9}$$

Universal quantum computation cannot be executed with operations acting only on one qubit. To construct a universal quantum computation, a two-qubit gate acting on two qubits is required. The two-qubit gate is specified by a 4×4 matrix:

$$\mathbf{W} = \begin{pmatrix} w_{00|00} & w_{00|01} & w_{00|10} & w_{00|11} \\ w_{01|00} & w_{01|01} & w_{01|10} & w_{01|11} \\ w_{10|00} & w_{10|01} & w_{10|10} & w_{10|11} \\ w_{11|00} & w_{11|01} & w_{11|10} & w_{11|11} \end{pmatrix}. \tag{10}$$

When this two-qubit gate acts on the k-th and l-th $(l > k)$ qubits, its action in the tensor product space of n qubits is:

$$W_{kl} = \sum_{i_1 \dots i_n} \sum_{j_k, j_l} u_{i_k i_l | j_k j_l} |i_1 \dots i_k \dots i_l \dots i_n\rangle \langle i_1 \dots j_k i_{k+1} \dots j_l i_{l+1} \dots i_n|. \tag{11}$$

By appropriately applying the two-qubit gate, any unitary transformation can be constructed. It is also known that any two-qubit gate can be approximately constructed from the product of specific basic gates, T gate, H gate, CNOT gate, which are given by respectively:

$$H = \frac{1}{\sqrt{2}} \begin{pmatrix} 1 & 1 \\ 1 & -1 \end{pmatrix}, \quad T = \begin{pmatrix} e^{-i\pi/8} & 0 \\ 0 & e^{i\pi/8} \end{pmatrix}, \quad \text{CNOT} = \begin{pmatrix} 1 & 0 & 0 & 0 \\ 0 & 1 & 0 & 0 \\ 0 & 0 & 0 & 1 \\ 0 & 0 & 1 & 0 \end{pmatrix}. \tag{12}$$

However, in this study, we do not decompose the two-qubit gate into $\{T, H, \text{CNOT}\}$, and we take the two-qubit gate as a basic unit. A quantum algorithm starts from the quantum state of n initialized qubits,

$$|0^n\rangle = \begin{pmatrix} 1 \\ \vdots \\ 0 \end{pmatrix} \tag{13}$$

and constructs a quantum circuit consisting of two-qubit gates according to the problem to be solved:

$$\mathcal{C} = \prod_{m=1}^{M} W^{(m)}_{k_m l_m} \tag{14}$$

to obtain the output quantum state $|\psi\rangle = \mathcal{C}|0^n\rangle$. When a measurement is performed on this quantum state, a certain n-bit string $b \in \{0, 1\}^n$ is obtained with probability

$$p_b = |\langle b|\psi\rangle|^2 \tag{15}$$

As will be explained later in the quantum kernel method, if there are two quantum circuits $\mathcal{C}$ and $\mathcal{C}'$, the square of the absolute value of the inner product between the quantum states $\mathcal{C}|0^n\rangle$ and $\mathcal{C}'|0^n\rangle$ generated by these can be written as

$$p_{0\dots0} = |\langle 0^n|(\mathcal{C}')^\dagger \mathcal{C}|0^n\rangle|^2 \tag{16}$$

From this, we can define a new quantum circuit $\mathcal{D} := (\mathcal{C}')^\dagger \mathcal{C}$, and estimate the probability of obtaining all zeros to know the value of the inner product from the quantum computer.

1.2 Automatic Quantum Circuit Encoding (AQCE) procedure

We here give a detailed explanation of the AQCE algorithm.

Assuming that the quantum circuit $\mathcal{C}$ is composed of M 2-qubit gates,

$$\mathcal{C} = \prod_{m=1}^{M} \mathcal{U}_m = \mathcal{U}_1\mathcal{U}_2 \dots \mathcal{U}_M, \tag{17}$$

With respect to the mth gate, F can be rewritten as follows:

$$F_m = \langle \Phi_{m-1} | \mathcal{U}_m^\dagger | \Psi_{m+1} \rangle . \tag{18}$$

Here, the quantum states $|\Phi_m\rangle$ and $|\Psi_m\rangle$ are defined, respectively, by

$$|\Psi_m\rangle = \prod_{k=m}^{M} \mathcal{U}_k^\dagger |\Psi\rangle = \mathcal{U}_m^\dagger \mathcal{U}_{m+1}^\dagger \dots \mathcal{U}_M^\dagger |\Psi\rangle , \tag{19}$$

$$\langle \Phi_m | = \langle 0 | \prod_{k=1}^{m} \mathcal{U}_k^\dagger = \langle 0 | \mathcal{U}_1^\dagger \mathcal{U}_2^\dagger \dots \mathcal{U}_m^\dagger. \tag{20}$$

Then, in terms of trace formula, F_m can be rewritten as

$$F_m = \mathrm{Tr}[|\Psi_{m+1}\rangle \langle \Phi_{m-1}| \mathcal{U}_m^\dagger]. \tag{21}$$

If we denote $\mathbb{I}_m = i, j$ as the indices of the qubits on which $\mathcal{U}_m$ acts, we can introduce the fidelity tensor operator $\mathcal{F}_m$

$$\mathcal{F}_m = \mathrm{Tr}_{\mathbb{I}_m}[|\Psi_{m+1}\rangle \langle \Phi_{m-1}|], \tag{22}$$

by which we can obtain F_m as

$$F_m = \mathrm{Tr}_{\mathbb{I}_m}[\mathcal{F}_m \mathcal{U}_m^\dagger], \tag{23}$$

Furthermore, if we consider $\mathbf{F}_m$ and $\mathbf{U}_m$ as the matrix representations of $\mathcal{F}_m$ and $\mathcal{U}_m$, respectively, we obtain

$$F_m = \mathrm{tr}[\mathbf{F}_m \mathbf{U}_m] \tag{24}$$

The singular value decomposition of $\mathbf{F}_m$ provides $\mathbf{F}_m = \mathbf{XDY}$, where $\mathbf{X}, \mathbf{Y}$ are unitary matrices, and $\mathbf{D}$ is a diagonal matrix with non-negative real diagonal elements d_n. Then, we have

$$F_m = \mathrm{tr}[\mathbf{XDYU}_m^\dagger] = \mathrm{tr}[\mathbf{DZ}] = \sum_{n=0}^{2^2-1} d_n [\mathbf{Z}]_{nn}, \tag{25}$$

where $\mathbf{Z} = \mathbf{Y}\mathbf{U}_m^\dagger \mathbf{X}$. This leads

$$|F_m| = \left| \sum_{k=0}^{2^2-1} d_n [\mathbf{Z}]_{nn} \right| \leq \sum_{k=0}^{2^2-1} d_n \left| [\mathbf{Z}]_{nn} \right| . \tag{26}$$

Given that $\mathbf{Z}$ is a unitary matrix, the absolute value of F_m is maximized when $\mathbf{Z}$ is an identity matrix. Therefore, the matrix $\mathbf{U}_m$ that maximizes the absolute value of F_m is given by

$$\mathbf{U}_m = \mathbf{X}\mathbf{Y}. \tag{27}$$

Using this, the quantum circuit is optimized through the following steps:

1. Set $m = 1$.
2. Compute the representation matrix $\mathbf{F}_m^{(k)}$ of the fidelity tensor $\mathcal{F}_m^{(k)} = \mathrm{tr}_{\mathbb{I}_k}[|\Psi_{m+1}\rangle \langle \Phi_{m-1}| \mathcal{U}_m]$ for all possible pairs $\mathbb{I}_k$ of indices.
3. Perform singular value decomposition $\mathbf{F}_m^{(k)} = \mathbf{X}^{(k)}\mathbf{D}^{(k)}\mathbf{Y}^{(k)}$ for all $\mathbf{F}_m^{(k)}$, and calculate $S^{(k)} = \sum_{n=0}^{3} [\mathbf{D}^{(k)}]_{nn}$.
4. Find k^* such that S_k^* is maximized.
5. Set $\mathbf{U}_m = \mathbf{X}^{(k^*)}\mathbf{Y}^{(k^*)}$, and apply the operator $\mathcal{U}_m$ specified by $\mathbf{U}_m$ acting on $\mathbb{I}_{k^*}$.
6. If $m < M$, increment m by 1 and return to step 2. If $m = M$, terminate the process.

By following these steps, the structure of the quantum circuit, including which qubits the two-qubit gates should operate on, is optimized while autonomously constructing a quantum circuit that encodes the desired quantum state. The fidelity of the embedded quantum state changes depending on the number of gates M employed in the circuit $\mathcal{C}$.

The AQCE algorithm combines this circuit optimization step with a gate addition step to optimize the circuit while deepening it. In this work, we add new δ two-qubit gates until $M < M_{\mathrm{max}}$ or $F < F_{\mathrm{target}}$.

1.3 Support Vector Machines

Support Vector Machines (SVM) are supervised learning classifiers that learn a decision boundary between two classes of elements. We define the training data as $TR = \{\vec{x}^{(i)}, \vec{y}^{(i)}\}_{i=1:N} \in X \times Z_2$ and test data $TS = \{\vec{x}^{(i)}, \vec{y}^{(i)}\}_{i=1:M} \in X \times Z_2$, generally with $TS \cap TR = \emptyset$, where the two sets are assumed to have been generated by the same or highly similar distribution. SVMs solve a convex optimization problem on TR to deliver a classifier for TS with a trade-off between an accurate performance for the true labels and maximization of the orthogonal distance between the classes. The classification boundary is generally highly non-linear, therefore the data is first mapped with a feature function $\phi : X \to F$ to a higher dimensional space (feature space, in the quantum case the Hilbert Space), with a scalar product $\langle , \rangle$. In this space, the SVM is a linear classifier which aims at maximizing the margin between decision boundary and data points:

$$\arg\max_{w,b} \left\{ \frac{1}{||w||} \min_n [t_n(\langle w, \phi(\vec{x}^{(n)}) \rangle + b)] \right\} \tag{28}$$

Here t_n are target class values for the elements $\vec{x}^{(i)} \in TR$, the $\frac{1}{||w||}$ is just a scaling factor to avoid dependence on n. As an intuition, $\vec{y}(\vec{x}^{(n)}) = \langle w, \phi(\vec{x}^{(n)}) \rangle + b$ defines the classification in one of the two classes based on its positive or negative sign. Instead, $t_n(\langle w, \phi(\vec{x}^{(n)}) \rangle + b)$ is the distance from the decision surface of the problem (which then we want to maximize). In our case, we use a soft margin SVM, which means that $t_n y(\vec{x}^{(n)}) \geq 1 - \xi_n$, where ξ_n are slack variables that help to deal with overlapping class distributions giving a small penalty for misclassification. The previous formulation of 28 is too complex to solve, therefore it is generally converted to the equivalent *primal problem*:

$$\begin{aligned} \underset{w,b,\xi}{\text{minimize}} \quad & \frac{1}{2}||w||^2 + C\sum_{n=1}^{N} \xi_n \\ \text{subject to} \quad & t_n(\langle w, \phi(\vec{x}^{(n)}) \rangle + b) \geq 1 - \xi_n, \\ & \xi_n \geq 0 \end{aligned} \tag{29}$$

The "Kernel trick" consists in shifting this formulation to one where we don't have to compute the computationally demanding mapping Φ for every point, but instead just compute a Kernel function defined as $K(\vec{x}^{(i)}, \vec{x}^{(j)}) = \langle \phi(\vec{x}^{(i)}), \phi(\vec{x}^{(j)}) \rangle$. Follows the dual formulation:

$$\begin{aligned} \underset{\alpha_i \in R}{\text{maximize}} \quad & \sum_{n=1}^{N} \alpha_n - \frac{1}{2}\sum_{n=1}^{N}\sum_{m=1}^{N} \alpha_n \alpha_m t_n t_m K(\vec{x}^{(n)}, \vec{x}^{(m)}) \\ \text{subject to} \quad & 0 \leq \alpha_i \leq C, \\ & \sum_{m=1}^{N} \alpha_n t_n = 0 \end{aligned} \tag{30}$$

Solving the dual problem allows then to solve the primal and the relationships for the equivalence allows expressing the classification function as:

$$f(\vec{x}) = sgn\Big(\sum_{n=1}^{N} t_n \alpha_n K(\vec{x}^{(i)}, \vec{x})\Big), \vec{x} \in TS \tag{31}$$

2 Machine Learning models and details

2.1 Structured State Space Sequence model (S4)

The Structured State Space sequence model (S4) is a neural network based on state space models designed to handle long sequences efficiently [1]. It is the first model to solve the Path-X task in the Long Range Arena benchmarks [2] that requires the ability to handle complex long-range dependencies, which other previous methods including Transformer failed to learn.

2.2 Transformer

Transformer is a neural network model consisting of self-attention and fully-connected feed-forward network [3]. Although it was originally proposed for machine translation, Transformer and its variants are now popularly used in various tasks, including computer vision [4], and achieve state-of-the-art performance. Compared with recurrent neural networks (RNNs) or convolutional neural networks (CNNs), Transformer depends on less inductive biases and thus expected to learn them from data.

2.3 Long Short-Term Memory (LSTM)

Long Short-Term Memory (LSTM) is a type of recurrent neural network (RNN) and has been the standard neural network for sequence modeling for a long time [5]. Compared with the vanilla RNN, LSTM has additional gates to control information so that it can handle longer sequences. These gates avoid gradient vanishing or gradient explosion, which the vanilla model suffers from, enabling it to apply to complex sequence tasks, such as machine translation [6].

2.4 Experimental settings and models

In this section, we provide a comprehensive overview of the configuration details for the S4, Transformer, and LSTM models used in our experiments. We trained these models on the proposed datasets in the QASM format for 200 epochs.

The input QASMs were preprocessed by removing superfluous information such as headers and rounding elements of dense matrices to *one* decimal place, as illustrated in Figure 5 of the paper. Interestingly, rounding to two decimal places decreased the performance. Test accuracy of S4 dropped from 77.78% to 72.10% for mnist_784 classification at a fidelity of 95%.

```
# The original format:
DenseMatrix(2,0,0.541645,0,-0.038637,0, ... ,0.540171,0) q[0],q[1];

 # The preprocessed format:
 0.5, 0, 0, 0, ..., 0.5, q[0], q[1]
```

2.4.1 S4 Model

The S4 model was implemented using a JAX-based version available at https://srush.github.io/annotated-s4/. We adopted its "CIFAR-10 classification" configuration, modifying the number of epochs.

The hyperparameters used for training the S4 model are as follows:

- **Hidden size**: 512
- **Number of layers**: 6
- **Dropout rate**: 0.25
- **Optimizer**: AdamW (initial learning rate of 1.0×10^{-3} with cosine annealing with warmup and weight decay rate of 0.01)
- **Batch size**: 50

2.4.2 Transformer Model

We adopted a BERT-like Transformer architecture as a sequence classifier [7] using the Transformer Encoder module in PyTorch. Layer normalization was applied prior to self-attention and feedforward layers (pre-norm), and the GELU activation function was used as [3].

The following hyperparameters are used for training the Transformer models.

- **Hidden size**: 512
- **Number of heads**: 8
- **Number of layers**: 6
- **Dropout rate**: 0.10
- **Optimizer**: AdamW (initial learning rate of 1.0×10^{-5} with cosine annealing with warmup and weight decay rate of 0.01)
- **Batch size**: 128

We selected the initial learning rate from $\{1.0 \times 10^{-5}, 5.0 \times 10^{-5}, 1.0 \times 10^{-4}\}$ and weight decay from $\{0.01, 0.05, 0.1\}$ on mnist_786 with a fidelity of 80.

2.4.3 LSTM Model

We implemented the LSTM model using the LSTM module in PyTorch followed by a linear layer with the following hyperparameters.

- **Hidden size**: 512
- **Number of layers**: 2
- **Dropout rate**: 0.25
- **Optimizer**: AdamW (initial learning rate of 1.0×10^{-3} with cosine annealing with warmup and weight decay rate of 0.01)
- **Batch size**: 128

We selected the initial learning rate from $\{1.0 \times 10^{-4}, 1.0 \times 10^{-3}\}$ on mnist_786 with a fidelity of 80.

2.5 Experiments with different data preprocessing

We show here alternative experiments using a different data processing strategy from the QASM files testing on Transformer and LSTM. The real part of the DenseMatrix in QASM is truncated to three decimal places and arranged in a specific format.

```
# The original format:
DenseMatrix(2,0,0.541645,0,-0.038637,0, ... ,0.540171,0) q[0],q[1];

# is converted to:
0.541 -0.038 ... 0.54 q[0] q[1] _
```

The classification process involves the following steps:

- **Data Conversion:** If there are 25 DenseMatrix instances, they are juxtaposed to form a single sequence.
- **Vocabulary Building:** The vocabulary is built from the real part (truncated to three decimal places), $q[0]$, and the data delimiter, _. The following command can be used to build the vocabulary:

```
onmt_build_vocab -config config.yaml -n_sample 1000
```

- **Training:** The model can be trained using the following command:

```
onmt_train -config config.yaml
```

Table 1 Test accuracy of Transformer and LSTM with different preprocessing from before.

Dataset	Fidelity	Transformer	LSTM
MNIST-784	80	**58.84**	55.68
	90	**57.74**	55.14
	95	54.90	**55.68**
Fashion-MNIST	80	**62.70**	55.00
	90	**57.96**	55.54
	95	55.00	55.00
Kuzushiji-MNIST	80	**56.39**	55.00
	90	**55.12**	55.11
	95	**55.12**	55.00

2.5.1 relative Transformer Model

For the Transformer and LSTM models, we employed OpenNMT-py [8]. The specific configuration for the Transformer model is detailed below:

- **Word vector size**: 512
- **Hidden size**: 512
- **Number of layers**: 6
- **Feed-forward size (transformer_ff)**: 2048
- **Number of heads**: 8
- **Accumulation count (accum_count)**: 8
- **Optimizer**: Adam (Beta1: 0.9, Beta2: 0.998)
- **Decay method**: Noam
- **Learning rate**: 2.0
- **Maximum gradient norm (max_grad_norm)**: 0.0
- **Dropout rate**: 0.1
- **Label smoothing**: 0.1

2.5.2 relative LSTM Model

The LSTM model was constructed using the following components, each with their respective hyperparameters:

- **RNNEncoder**: LSTM encoder with 500 hidden units
- **RNNDecoder**: Stacked LSTM decoder with two LSTM cells (First cell: 1000 hidden units, Second cell: 500 hidden units)
- **GlobalAttention**: Implemented in the model
- **Generator**: Linear layer with 500 units for output generation

- **Word vector size**: 500
- **Hidden size**: 500
- **Number of layers**: 2
- **Optimizer**: SGD
- **Accumulation count**: 1
- **Learning rate**: 1.0
- **Maximum gradient norm**: 5
- **Dropout rate**: 0.3

3 Additional Information

4 Dataset license

We release MNISQ dataset with the following license:
CC BY-SA 4.0
https://creativecommons.org/licenses/by-sa/4.0/

5 Metadata

Structured metadata have been added to the Zenodo record associated with the dataset https://doi.org/10.5281/zenodo.19656638. For transparency and ease of reuse, the structured metadata are also provided in the GitHub repository at https://github.com/FujiiLabCollaboration/MNISQ-quantum-circuit-dataset/blob/main/metadata.

6 GitHub url

The dataset and the experiments are accessible on the GitHub URL: https://github.com/FujiiLabCollaboration/MNISQ-quantum-circuit-dataset.

References

[1] Gu, A., Goel, K., Ré, C.: Efficiently Modeling Long Sequences with Structured State Spaces (2022). https://doi.org/10.48550/arXiv.2111.00396

[2] Tay, Y., Dehghani, M., Abnar, S., Shen, Y., Bahri, D., Pham, P., Rao, J., Yang, L., Ruder, S., Metzler, D.: Long range arena : A benchmark for efficient transformers. In: International Conference on Learning Representations (2021). https://openreview.net/forum?id=qVyeW-grC2k

[3] Vaswani, A., Shazeer, N., Parmar, N., Uszkoreit, J., Jones, L., Gomez, A.N., Kaiser, Ł., Polosukhin, I.: Attention is all you need. In: Guyon, I., Luxburg, U., Bengio, S., Wallach, H., Fergus, R., Vishwanathan, S.V.N., Garnett, R. (eds.) Advances in Neural Information Processing Systems, vol. 30. Curran Associates, Inc., Red Hook, NY, USA (2017). https://proceedings.neurips.cc/paper_files/paper/2017/file/3f5ee243547dee91fbd053c1c4a845aa-Paper.pdf

[4] Dosovitskiy, A., Beyer, L., Kolesnikov, A., Weissenborn, D., Zhai, X., Unterthiner, T., Dehghani, M., Minderer, M., Heigold, G., Gelly, S., Uszkoreit, J., Houlsby, N.: An image is worth 16x16 words: Transformers for image recognition at scale. In: International Conference on Learning Representations (2021). https://openreview.net/forum?id=YicbFdNTTy

[5] Hochreiter, S., Schmidhuber, J.: Long short-term memory. Neural Computation **9**(8), 1735–1780 (1997) https://doi.org/10.1162/neco.1997.9.8.1735

[6] Sutskever, I., Vinyals, O., Le, Q.V.: Sequence-to-sequence learning with neural networks. Advances in Neural Information Processing Systems **27**, 3104–3112 (2014)

[7] Devlin, J., Chang, M.-W., Lee, K., Toutanova, K.: Bert: Pre-training of deep bidirectional transformers for language understanding. arXiv preprint arXiv:1810.04805 (2018) https://doi.org/10.48550/arXiv.1810.04805

[8] Klein, G., Kim, Y., Deng, Y., Senellart, J., Rush, A.: OpenNMT: Open-source toolkit for neural machine translation. In: Proceedings of ACL 2017, System Demonstrations, pp. 67–72. Association for Computational Linguistics, Vancouver, Canada (2017). https://www.aclweb.org/anthology/P17-4012
